# The Effect of Healthy Diet on Cognitive Performance Among Healthy Seniors – A Mini Review

**DOI:** 10.3389/fnhum.2020.00325

**Published:** 2020-08-11

**Authors:** Blanka Klimova, Szymon Dziuba, Anna Cierniak-Emerych

**Affiliations:** ^1^Department of Applied Linguistics, Faculty of Informatics and Management, University of Hradec Králové, Hradec Králové, Czechia; ^2^Faculty of Engineering and Economics, Wrocław University of Economics, Wrocław, Poland

**Keywords:** diet, nutrients, dietary patterns, seniors, cognition, cognitive decline, prevention

## Abstract

At present, a healthy diet appears to be one of the suitable strategies in slowing down cognitive decline in the process of aging. A number of evidence-based studies confirm its efficacy, safety, and cost-effectiveness. The aim of this mini review is to evaluate and describe recent randomized clinical and cohort studies exploring the effect of healthy diet on cognitive performance among healthy seniors, as well as to update the existing information on this research issue. For these reasons, the authors reviewed full-text, peer-reviewed journal articles written in English and available in Web of Science and PubMed between September 2017 and February 2020. Altogether nine original studies were detected. The results indicate that healthy diet and healthy diet components generally have a positive impact on the enhancement of cognitive functions. Furthermore, the findings reveal that dietary patterns, as well as single nutrients might have a significant effect on specific cognitive domains, such as memory in general, episodic memory, or processing speed. It also seems that a strict adherence to the dietary patterns and a higher diet variety have a more significant effect on the improvement of cognitive functions. Nevertheless, there seem to be gender differences in dietary behavior. More recently, personalized dietary interventions started to be used in delaying cognitive decline among healthy seniors. Therefore, more randomized control trials or N-of-1 trials should be performed in this research area in order to detect the most suitable dietary pattern or nutrients, which would, together with other modifiable lifestyle factors, contribute to the improvement of quality of life of the aging population.

## Introduction

A healthy diet is one of the modifiable risk factors in the fight against dementia, whose initial manifestation usually starts with deterioration of cognitive functions (Rakesh et al., 2017). Due to the increase in the elderly population worldwide, the spread of this neurological disorder is on the rise. Statistical data reveal that nowadays there are about 50 million people living with dementia and there are about 10 million new cases every year. The scholars estimate that by 2030, there will be 82 million people suffering from dementia and by 2050, this number will reach 152 million (WHO, 2020). At present, this debilitating syndrome cannot be cured (Klimova et al., 2016). Therefore, there is a sustainable effort to discover alternative, effective, safe, and inexpensive strategies which could contribute to the prevention of dementia and cognitive decline and strive to improve the quality of life of aging population (Livingston et al., 2017; Vauzour et al., 2017; Cremonini et al., 2019). Evidence suggests that healthy diet or nutrition may be one of these preventive strategies (Kita et al., 2018; Mazza et al., 2018; Chen et al., 2019; Dodd et al., 2019; Karstens et al., 2019; Soldevila-Domenech et al., 2019).

Individual nutrients, such as vitamins, polyunsaturated fatty acids (PUFAs), and flavonoids play an important role in the enhancement of cognitive performance among healthy older people (Cremonini et al., 2019; Dodd et al., 2019). In addition, single foods, such as avocados (Scott et al., 2017), berries (Whyte et al., 2018), or extra-virgin olive oil (Klimova et al., 2019) are connected with the delay of cognitive decline. For example, extra-virgin oil may have a neuroprotective effect and could positively prevent the development of dementia, especially Alzheimer’s dementia. Especially, its component, secoiridoid oleuropein seems to be responsible for this effectiveness (Klimova et al., 2019). The impact of extra-virgin olive oil on the short-term improvement of cognitive functions scores among older individuals rather than Mediterranean Diet (MedDiet) alone was also confirmed by Mazza et al. (2018).

Recently more attention has been paid to specific dietary patterns, including an appropriate combination of several nutrients which seem to have a bigger impact in this respect (Scarmeas et al., 2018; Solfrizzi et al., 2018; Karstens et al., 2019; Gu et al., 2020). However, as Mazza et al. (2018) state, it is the quality of the food that is significant in each dietary pattern, not the quantity. Furthermore, Smyth et al. (2015) expand that the higher diet quality in terms of a higher intake of healthy food choices is a significant potential method for lowering the global burden of cognitive decline. Wright et al. (2017) show that a higher diet quality is associated with better performance, especially in the area of verbal retention and memory, irrespective of race and poverty status. Moreover, personalized dietary counseling contributes to higher adherence to healthy diets, which is consequently connected with smaller cognitive decline and a lower risk of Alzheimer’s disease (van den Brink et al., 2019).

Currently, there are three basic dietary patterns: MedDiet, Dietary Approach to Stop Hypertension (DASH), and Mediterranean-DASH diet Intervention for Neurodegenerative Delay (MIND; Willett et al., 1995; Cremonini et al., 2019). The MedDiet pattern is characterized by fruits, vegetables, whole grains, olive oil, fish, nuts, legumes, moderate wine consumption, low consumption of processed foods, dairy products, red meat, and vegetable oils. This diet consists of the nutrients such as monounsaturated fatty acids, PUFAs, antioxidants (allium sulfur compounds, anthocyanins, beta-carotene-flavonoids, catechins, carotenoids, indoles, or lutein), vitamins (A, B1,6,9,12, D, E), minerals (magnesium, potassium, calcium, iodine, zinc, and selenium), which have a significant positive effect on pathological neurodegenerative processes such as oxidative stress, neuroinflammation, insulin resistance, or reduced cerebral blood flow (Scarmeas et al., 2006; Knight et al., 2016; Dinu et al., 2017).

Similarly, the DASH diet, which originated in the United States in 1990s, is rich in fruits, vegetables, fish, whole-cereal products, but also in low-fat dairy products, which contain nutrients such as potassium, calcium, lean proteins, minerals, and fiber (Steinberg et al., 2017; Cremonini et al., 2019; Challa et al., 2020). On the other hand, this dietary pattern reduces foods high in saturated fat and sugar (Appel et al., 1997). Apart from lowering cholesterol, saturated fats, and blood pressure, it has an effect on the improvement of cognitive functions among older people, provided they adhere to this diet for a longer time (Tangney et al., 2014; Berendsen et al., 2017).

The MIND patterns originated as a combination of the DASH and MedDiet to create a diet that would reduce the risk of dementia and slow down the neurodegeneration process, especially Alzheimer’s disease (Moris, 2016). It includes the 10 brain-healthy food groups (green leafy vegetables, other vegetables, nuts, berries, beans, whole grains, seafood, poultry, olive oil, and wine) and the five unhealthy food groups (red meats, butter and stick margarine, cheese, pastries, and sweets, and fried/fast food), all of which prove to be connected with the prevention against dementia (Koch and Jensen, 2016; Cremonini et al., 2019). In comparison with the former dietary patterns, the MIND diet specifies green leafy vegetables and berries and relevant amounts of food components (Moris, 2016). Especially leafy vegetables have one of the most significant neuroprotective effects (Morris et al., 2015a). Research also indicates that the strongest associations between healthy dietary patterns and enhanced cognitive performance have been observed for the MIND diet (Morris et al., 2015b; Hosking et al., 2019).

Evidenced-based studies confirm that all three dietary patterns seem to have at least a moderate positive effect on cognitive performance among healthy seniors. However, there are also research studies which disagree with these positive impacts. They claim that dietary interventions, especially dietary monotherapies, do not have any effect on the improvement of cognitive functions among healthy older population groups (Krause and Roupas, 2017; Akbaraly et al., 2019). But one thing is certain, the dietary approaches are non-invasive and have fewer or no side effects if compared to drug therapies (Klimova and Kuca, 2018).

The aim of this review is to evaluate and describe the recent randomized clinical and cohort studies exploring the effect of healthy diet on cognitive performance among healthy seniors, as well as to update the existing information on this research issue.

## Methods

The authors adhered to the PRISMA guidelines for this part. They systematically reviewed full-text, peer-reviewed journal articles written in English and available in Web of Science and PubMed between September 2017 and February 2020. The reason for the selection of this period was that a comprehensive and systematic review (Chen et al., 2019) had been performed on this topic before this period. Only randomized clinical trials and cohort studies were included. The research topic had to focus on the effect of diet on cognitive performance among healthy seniors. The selected studies involved groups where the population had to be cognitively unimpaired and 55 years old or over. Categorical searches were conducted using the following keywords: diet AND cognition AND older people, diet AND cognition AND elderly, diet AND cognition AND seniors, nutrition AND cognition AND older people, nutrition AND cognition AND elderly, nutrition AND cognition AND seniors.

The terms used were queried using AND to combine the keywords listed and using OR to remove search duplication where possible. In addition, a backward search was also performed, i.e., references of detected studies were evaluated for relevant research studies that authors might have missed during their search. In addition, a google search was conducted in order to detect unpublished (gray) literature. The authors performed an independent quality assessment of these studies. They read the articles to assess eligibility and to determine the quality. The basic quality criteria were adequately described study design, participant characteristics, control conditions, outcome measures, and key findings, with special focus on statistically significant differences (Table 1). The authors selected these basic quality criteria using Health Evidence Quality Assessment Tool for review articles.

**TABLE 1:** Overview of the findings from nine detected studies on the impact of diet/nutrition on cognitive functions among healthy older people.

Author, type of study, country, follow-up	Study sample	Type of diet and its intervention	Main outcome measures and assessed cognitive domains	Findings
Chai et al. (2019) (RCT) United States Follow-up: None.	37 healthy seniors, age range: 65–80 years, 17 males, 20 females. 20 participants were in the intervention group and 17 in the control group.	The intervention group received two cups of Montmorency tart cherry juice per day for 12 weeks.	Montreal Cognitive Assessment Test, CANTAB assessing episodic visual memory and new learning, attention, speed of response and movement, working memory, and a subjective memory questionnaire.	The intervention group had higher contentment with memory scores (mean difference of 2.7; 95% CI: 1.2 to 4.2; *p* = 0.02), especially in spatial working memory, as well as in visual sustained attention.
Danthiir et al. (2018) (RCT) Australia Follow-up: None.	391 participants, age range: 65–90, 195 seniors in the intervention group, 196 seniors in the control group.	The intervention group received 1720 mg DHA and the control group 600 mg eicosapentaenoic acid or low-polyphenolic olive oil daily in the form of capsules for 18 months.	MMSE, Sternberg’s number and letter memory scanning, Simon task, Stroop test, Australian Short-Form Health Survey, the Yale Physical Activity Scale, the Dietary Questionnaire, Polyunsaturated Fatty Acid Questionnaire, Late Life function and Disability Instrument, Cognitive Failure Questionnaire, the Prospective and Retrospective Memory Questionnaire, the Satisfaction with Life Scale, the Positive and Negative Affect Scale, the Center for Epidemiological Studies Depression Scale, the Nottingham Sleep Survey.	Daily supplementation with 2.3 *g* DHA-rich fish oil for 18 months did not maintain or improve cognitive performance. A small negative main effect was found on psychomotor speed in men (intervention = -0.02, 95% CI: -0.04 to 0.00; *d* = 0.24, *P* = 0.03).
Karstens et al. (2019) (Cohort study – cross-sectional) United States.	82 healthy seniors at the age of 68.8 years, 50% males and 50% females; participants were divided into High and Low (median split) adherence groups.	Adherence to MedDiet.	Block Food Frequency Questionnaire 2005, standardized cognitive assessment battery of tests (i.e., the California Verbal Learning Tests, Trail Making Tests – Part A, Part B, and the Wechsler Test of Adult Reading), MRI, T1-weighted images, FreeSurfer 6.0 segmentation pipeline. Assessed cognitive domains: learning and memory, information processing and executive functioning.	The High MedDiet group was better at learning and memory performance [β = 0.52, SE = 0.21, *t*(74) = 2.53, *P* = 0.01, *d* = 1.23].
Kuroda et al. (2019) (RCT) Japan Follow-up: None.	52 healthy seniors, randomly assigned into a UHHPBR group (27 subjects) and polished WR group (25 subjects), mean age: 72.9 + 0.8 years, 25 males, 27 females.	100 g of UHHPBR per day for 2 years (intervention group) and 100 g of white rice (control group).	Revised Hasegawa’s Dementia Scale, Mini-Mental State Examination, FAB, Cognitive Assessment for Dementia, iPad version (CADi) assessed cognitive function.	A 2-y oral consumption of UHHPBR increases information processing speed (the total FAB score was greater in the UHHPBR group than in the WR group, *p* = 0.062, and improves apathy (*p* = 0.012) among healthy seniors.
Marseglia et al. (2018) (RCT) China, France, Italy, Netherlands, Poland, Sweden, United Kingdom Follow-up: 1 year.	1,279 healthy seniors, age range: 65–79 years, from five European centers. They were randomly assigned into a control group (638) and an intervention group (641).	Intervention group: individually tailored Mediterranean-like dietary advice (NU-AGE diet); control group – habitual diet. After the baseline assessment, both groups completed a seven-day food record for seven consecutive days over 2 weeks and had an interview with a trained dietician to review the records. The intervention group had a monthly education telephone calls and face-to-face meetings with a dietician.	CERAD – Neuropsychological Battery, MMSE, Babcock Story Recall Test, Pattern Comparisons, Digit Cancelation, Trail Making Tests, Word List Memory, 15-items Boston Naming Test, Constructional Praxis Test, Category fluency. Assessed cognitive domains: global cognition, perceptual speed, executive function, episodic memory, verbal abilities, and constructional praxis.	Subjects with higher adherence to the NU-AGE diet experienced considerable improvements in global cognition [β 0.20 (95%CI 0.004, 0.39), *p*-value = 0.046] and episodic memory [β 0.15 (95%CI 0.02, 0.28), *p*-value = 0.025] after 1 year, compared to those adults with lower adherence to NU-AGE diet. Both groups of subjects improved in global cognition and in all cognitive domains after 1 year.
Milte et al. (2019) (Cohort study – prospective) Australia.	617 participants, age range: 55–65 years.	Different food choices (diet variety). The study lasted from 2010–2014.	A postal survey including a 111-item Food Frequency Questionnaire, dietary guideline index (DGI-2013), Telephone Interview of Cognitive Status (TICS-m), which assessed cognitive function.	There were no associations between diet quality in 2010 and cognitive function in 2014. However, participants who reported higher dietary variety
				(*B* = 0.28, 95% CI 0.03, 0.52) and women who reported “sometimes” adding salt to food after cooking (*B* = 0.98, 95% CI 0.25, 1.71) in 2010 displayed better cognitive function in 2014. In 2014, usual consumption of higher fiber bread choices in the total sample (*B* = 1.32, 95% CI 0.42, 2.23), and higher diet quality (*B* = 0.03, 95% CI 0.00, 0.07) and greater fluid consumption (*B* = 0.14, 95% CI 0.01, 0.27) in men were all associated with better cognitive function.
Moran et al. (2018) (RCT) Ireland Follow-up: at 3, 6 months.	37 older people, age range: 68–83 years, 20 seniors in the intervention group and 17 in the control group, 18 males and 19 females.	The intervention group received (Smartfish), containing omega-3 polyunsaturated fatty acids (PUFAs), vitamin D, resveratrol, and whey protein. The control group placebo – 200 ml palatable, pomegranate and apple flavored juice formulations only. It lasted 6 months.	Time-Up and Go test, Cognitive Failures Questionnaire, Trail Making Test, Auditory Verbal Learning Test, Stroop test, Color and Color-Word tasks, Controlled Oral Word Association test, Digit Span test.	Only a limited beneficial impact was shown, especially for Stroop Color-Word Time (*p* < 0.05) in the intervention group. The intervention group demonstrated reduced task completion time at three- and six-month follow-ups, indicating enhanced performance.
Okubo et al. (2017) (cohort study – cross-sectional) Japan.	635 healthy elderly at the age of 69–71 years.	Different dietary patterns (the “Plant foods and fish” – high intakes of green and other vegetables, soy products, seaweeds, mushrooms, potatoes, fruit, fish, and green tea; “Rice and miso soup”; and “Animal food” – seasonings, shellfish, chicken, red meats, fish, seafood, and processed meats). The whole study lasted from 2010–2012.	A self-administered diet history questionnaire, Montreal Cognitive Assessment Test assessing nine cognitive domains: attention, concentration, executive functions, memory, language, visuoconstructional skills, conceptual thinking, calculation and attention.	The “Plant foods and fish” pattern, characterized by, was significantly associated with a higher MoCA-J score [MoCA-J score per one-quartile increase in dietary pattern: β = 0.56 (95% CI: 0.33, 0.79), *P* for trend <0.001]. In contrast, neither the “Rice and miso soup” nor the “Animal food” pattern was related to cognitive function.
Whyte et al. (2018) (RCT) United Kingdom Follow-up: 6 months.	122 older people, age range: 65–80 years. Participants were divided into four groups (3 intervention groups – 92 people) and one placebo control group – 30.	30 people received a whole wild blue-berry powder at 500 mg and 31 subjects 1000 mg and 31 people a purified extract at 100 mg (WBE111). The intervention lasted 6 months.	Rey’s Auditory Verbal Learning Task and Corsi Blocks Task were used for measuring verbal episodic memory and visual memory.	The findings show that a 3-month intervention with WBE111 can enhance episodic memory performance among healthy seniors (*p* < 0.05).

*CANTAB, Cambridge Neuropsychological Test Automated Battery; CERAD, Consortium to Establish a Registry for Alzheimer’s Disease; CHAS, Canadian Healthy Aging Study; DHA, docosahexaenoic acid; FAB, Frontal Assessment Battery; MedDiet, Mediterranean Diet; MMSE, Mini-Mental State Examination; MRI, magnetic resonance imaging; NU-AGE, New dietary strategies addressing the specific needs of elderly population for an healthy aging in Europe; TICS-m, Telephone Interview of Cognitive Status; UHHPBR, ultra-high hydrostatic pressurizing brown rice; WR, white rice.*

The primary outcome of this review study is:

• To evaluate and describe the recent randomized clinical trials (RCT) and cohort studies exploring the effect of healthy diet on cognitive performance among healthy seniors,
• To update the existing information on this research issue.

The authors identified a total of 411 studies in the databases described above. 236 articles were detected in PubMed and 175 articles were found in the Web of Science. Another 8 articles were detected from additional sources, most often from the references of the identified studies. After removing duplicates and titles/abstracts unrelated to the research topic, 141 English-written studies remained. Of these, only 43 articles were relevant for the research topic since the other studies (98) after screening their content were found irrelevant. 43 studies were thus investigated in full and they were considered against the following inclusion and exclusion criteria. The inclusion criteria were as follows:

• The review period was restricted by September 1, 2017 up to February 29, 2020.
• The studies had to be peer-reviewed, full-text and written in English.
• Only randomized controlled trials and cohort studies were involved into the review.
• The primary outcome was aimed at the effect of healthy diet on cognitive performance among healthy seniors.
• The participants had to be at the age 55+ without any cognitive impairment. The exclusion criteria were as follows:
• The study protocols, e.g., (Mumme et al., 2019), multi-domain intervention studies, e.g., (Lehtisalo et al., 2017; Rosenberg et al., 2018), the studies with different age range of seniors, e.g., (Kita et al., 2018), the studies with cognitively impaired seniors, e.g., (Baleztena et al., 2018; Mazza et al., 2018), and cross-over randomized control study (Dodd et al., 2019), the review studies, e.g., (Vauzour et al., 2017; Chen et al., 2019; Cremonini et al., 2019; Soldevila-Domenech et al., 2019).

**FIGURE 1:**
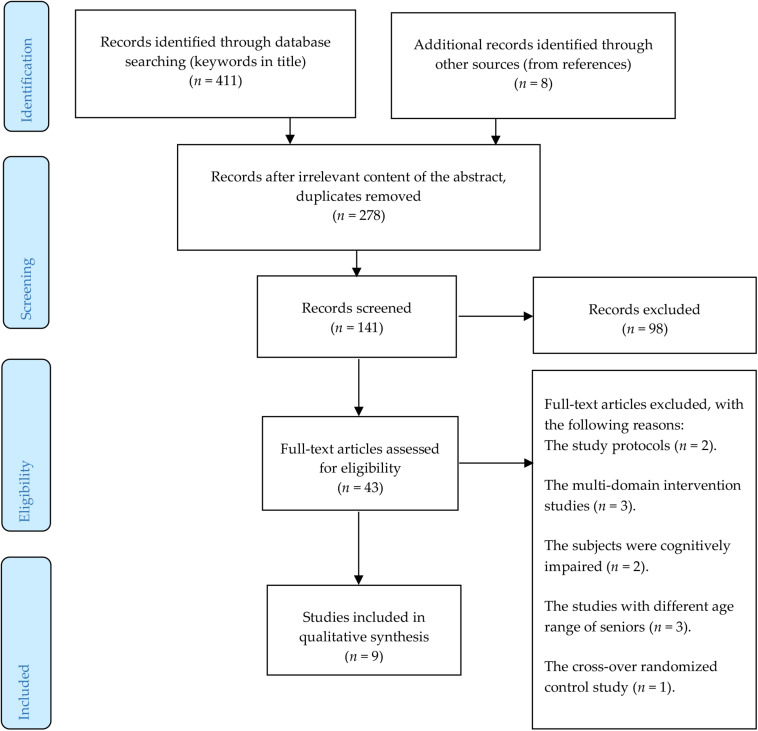
An overview of the selection procedure.

Considering the above described criteria, nine articles were eventually included into the final analysis. Figure 1 below describes the selection procedure.

## Results

Altogether nine original studies were detected in the databases described above. Six studies (Danthiir et al., 2018; Marseglia et al., 2018; Moran et al., 2018; Whyte et al., 2018; Chai et al., 2019; Kuroda et al., 2019) were RCT and three studies (Okubo et al., 2017; Milte et al., 2019; Karstens et al., 2019) were cohort studies. Two studies (Chai et al., 2019; Karstens et al., 2019) originated in United States, two (Okubo et al., 2017; Kuroda et al., 2019) in Japan, two in Australia (Danthiir et al., 2018; Milte et al., 2019), one in Ireland (Moran et al., 2018), one in United Kingdom (Whyte et al., 2018), and one study was an European multicenter study (Marseglia et al., 2018). Only two studies had a follow-up period. The main research topics focused on the explanation of the impact of individual nutrients [i.e., tart cherry juice, (DHA)-rich fish oil, blueberry powder/extract, and rice] or dietary patterns interventions (i.e., MedDiet, NU-AGE, Smartfish^®^) on cognitive performance on healthy seniors. The cohort studies usually lasted between 3–4 years. The length of RCT was between 3 and 24 months. The number of participants in RCT ranged between 37 and 1,279 and in the cohort studies between 82 and 635 healthy older individuals. The main outcome measures included a battery of standardized cognitive tests. In addition, the cohort studies usually were based on questionnaire surveys, interviews, or dietary guideline index. The assessment of cognitive domains focused especially on memory, attention, processing speed and executive functions.

The findings indicate that the described dietary and single nutrient interventions had a positive effect on global cognition (Marseglia et al., 2018; Moran et al., 2018), specifically on memory performance (Chai et al., 2019; Karstens et al., 2019), processing speed (Kuroda et al., 2019), and episodic memory (Okubo et al., 2017; Marseglia et al., 2018; Whyte et al., 2018). One study (Moran et al., 2018) reported a minor effect, and that concerned a reduced task completion time in the follow-up periods, revealing improved performance. Only one study (Danthiir et al., 2018) reported zero effect on the improvement of cognitive performance among seniors who were provided with a daily dose of fish oil for 18 months. Table 1 below gives a summary of the key results from the selected articles. The findings are outlined in alphabetical order by their first author.

## Discussion

The results described above indicate that healthy diet and healthy diet components generally have a positive impact on the enhancement of cognitive functions. This is in line with other studies on this research topic (Vauzour et al., 2017; Calil et al., 2018; Kita et al., 2018; Mazza et al., 2018; Solfrizzi et al., 2018; Chen et al., 2019; Cremonini et al., 2019; Dodd et al., 2019). Furthermore, the findings reveal that healthy diet and healthy diet components might have effects on specific cognitive domains, such as memory in general (Chai et al., 2019; Karstens et al., 2019), episodic memory (Okubo et al., 2017; Marseglia et al., 2018; Whyte et al., 2018), or processing speed (Kuroda et al., 2019). Better memory performance influenced by targeted nutrition interventions was also mentioned in previous research studies, e.g., Kulzow et al. (2016), whose intervention was based on the omega-3 fatty acid supplementation. However, in our detected study (Moran et al., 2018), the authors found only a small benefit on cognitive performance when supplying their intervention group with (Smartfish^®^), containing omega-3 PUFAs, vitamin D, resveratrol, and whey protein. The smaller effect might have been caused by a slightly older age group of participants. In our mini review, especially the consumption of vitamins (e.g., vitamin B6 and B12) in ultra-high hydrostatic pressurizing brown rice (Kuroda et al., 2019) and the consumption of flavonoids contained in tart cherry juice (Chai et al., 2019), plant foods and fish diet (Okubo et al., 2017), and blueberry extract (Whyte et al., 2018) were associated with enhanced cognitive performance. As Ayaz et al. (2019) state, the mechanisms of flavonoids have been shown to be mediated through the inhibition of cholinesterases including acetylcholinesterase (AChE), and butyrylcholinesterase (BChE), β-secretase (BACE1), free radicals and modulation of signaling pathways that are implicated in cognitive and neuroprotective functions.

It also seems that a higher adherence to the dietary patterns (Karstens et al., 2019; van den Brink et al., 2019), as well as a higher diet variety (Milte et al., 2019) have a more significant effect on the improvement of cognitive functions.

The findings also reveal that there might be gender differences in dietary behavior and its impact on the enhancement of cognitive functions. In their research study, Milte et al. (2019) state that females who sometimes added salt to food after cooking (B = 0.98, 95% CI 0.25, 1.71) in 2010 displayed better cognitive function in 2014. On the contrary, males who usually added salt to their food during cooking displayed poorer cognitive function (B = −1.37, 95% CI −2.39, −0.35). However, in 2014, usual consumption of higher fiber bread choices in the total sample (B = 1.32, 95% CI 0.42, 2.23), and higher diet quality (B = 0.03, 95% CI 0.00, 0.07) and greater fluid consumption (B = 0.14, 95% CI 0.01, 0.27) among men were connected with better cognitive performance. The gender differences in dietary behavior have been reflected in other studies (e.g., Okubo et al., 2017; Fard et al., 2019). In addition, the findings show (Okubo et al., 2017) that women do more care about their choice of foods since the dietary approach (plant foods and fish) enhancing cognitive performance was typical of women, usually possessing a higher educational degree, going outdoor frequently and avoiding smoking and drinking alcohol.

Furthermore, the results indicate that most of the studies now focus on dietary patterns rather than on single nutrients because research shows that the multi-nutrient dietary interventions, in which individual nutrients interact with each other, such as MedDiet or MIND diet have a far greater impact on slowing down cognitive decline (Okubo et al., 2017; Marseglia et al., 2018; Chen et al., 2019; Karstens et al., 2019; Milte et al., 2019).

In addition, personalized dietary interventions which address the impact of a healthy diet on cognitive performance among healthy older people have emerged. This was successfully reflected in the study by Marseglia et al. (2018). The findings of this study show that the seniors in the intervention group receiving individual tailored-made Mediterranean-like dietary advice (NU-AGE diet) experienced considerable improvements in global cognition [β 0.20 (95% CI 0.004, 0.39), *p*-value = 0.046] and episodic memory [β 0.15 (95% CI 0.02, 0.28), *p*-value = 0.025] after 1 year when compared with the control group. In fact, the trend toward a more personalized medicine is nowadays also supported with the emergence of new technological devices, which can educate and provide expert advice to seniors about the lifestyle modification strategies, such as appropriate dietary patterns in order to slow down their age-related cognitive decline (Vanoh et al., 2018). These personalized dietary interventions can then contribute to higher adherence to healthy diet as it has been already indicated in the section “Introduction.” Research goes even further and suggests conducting N-of-1 trials that would explore nutrition and cognitive performance in an individual. The reason is that each individual responds differently to dietary components, which are influenced by the interplay between various factors, such as environment, society, metabolism and genetics (Soldevila-Domenech et al., 2019).

The authors of this review also detected the research study (Danthiir et al., 2018) whose objective was to evaluate the use of docosahexaenoic acid-rich fish oil on reduction of cognitive decline in healthy seniors, which had zero effect. This finding is in line with an older Dutch RCT (van de Rest et al., 2008) that confirmed no effect of fish oil on cognitive performance in older subjects. On the contrary, a recent systematic review (Fard et al., 2019) on the effect of fish oil on various life conditions, such as the heart and cardiovascular system, the brain and visual function, inflammation and immune function and growth/Body Mass Index, report that fish oil seems to be effective in slowing the rate of cognitive decline cognitive function, especially in memory, of older people. Nevertheless, the authors also conclude that the results of the detected studies are inconsistent and there are gender and age specific differences.

The limitations of this review especially consists in methodological differences of the selected studies, including the type of the studies. Most of the detected studies focused on the assessment of both global cognition and individual cognitive domains. However, they used different types of tests assessing them. Furthermore, in some studies there were smaller sample sizes, short duration of RCT, and a lack of follow-up periods. There were also different types of interventions involved. The authors also reviewed only two databases, which might have excluded some of the peer-reviewed studies. All these insufficiencies might generate overestimated conclusions in this review study (Melby-Lervag and Hulme, 2016).

Overall, the findings of this review confirm that a healthy diet has a positive effect on slowing down cognitive decline among healthy seniors. However, as stated in the section “Introduction,” diet is only one of the lifestyle modifying factors. It should be employed together with other factors, such as physical activities or cognitive training, when it has even a higher effect on cognitive functions among seniors (Lehtisalo et al., 2017; Klimova and Valis, 2018; Rosenberg et al., 2018). Calder et al. (2018) report that there is growing evidence to encourage change in older people’s lifestyle, i.e., to make them aware of the importance of healthy diet, physical activities or cognitive training in order to maintain and/or enhance body composition, cognitive, mental health, and vascular health. They claim that lifestyle change at any phase of life might extend healthy lifespan. However, the earlier one starts, the more effective the changes will be.

Therefore, future research should focus on relevant dietary patterns on the enhancement of cognitive functions among healthy older individuals. This should be done particularly by joint multinational randomized controlled trials and N-of-1 trials that would detect the most suitable dietary pattern for this population groups, which would, together with other modifiable lifestyle factors, contribute to the improvement of quality of life of aging population. In addition, researchers should also find a compromise on outcome measures in order to achieve more reliable and valid results, which could be further compared and reviewed.

## Author Contributions

BK, SD, and AC-E wrote, read, and, agreed to the published version of the manuscript. All authors contributed to the article and approved the submitted version.

## Conflict of Interest

The authors declare that the research was conducted in the absence of any commercial or financial relationships that could be construed as a potential conflict of interest.
